# Toxic Epidermal Necrolysis Caused by Eribulin

**DOI:** 10.7759/cureus.61761

**Published:** 2024-06-05

**Authors:** Hirofumi Kawamoto, Natsuko Saito-Sasaki, Yu Sawada

**Affiliations:** 1 Dermatology, University of Occupational and Environmental Health, Kitakyushu, JPN

**Keywords:** case report, sarcoma, eribuin, drug eruption, toxic epidermal necrolysis (ten)

## Abstract

Eribulin, a chemotherapy drug classified as a microtubule inhibitor, is known to target cell microtubule structures, impeding cancer cell growth and spread. This paper discusses a rare case of toxic epidermal necrolysis (TEN) induced by eribulin in a patient with angiosarcoma, marking it as an uncommon adverse reaction. This patient developed severe mucosal and skin lesions after the third dose of eribulin. Laboratory tests and a skin biopsy confirmed the diagnosis of TEN. The patient responded well to steroid therapy, although skin eruptions reoccurred with further eribulin treatment. This case highlights the need for further study on the immunological effects of eribulin, especially concerning severe drug eruptions potentially related to its impact on microtubule dynamics and immune cell functions.

## Introduction

Eribulin is a chemotherapy drug classified as a microtubule inhibitor. It hinders the growth and spread of cancer cells by targeting their microtubule structures, leading to cell death and slowing disease progression [1]. While various adverse reactions to eribulin treatment have been documented [2], cutaneous adverse reactions have not been reported. This paper presents a case of toxic epidermal necrolysis (TEN) triggered by eribulin in a patient with angiosarcoma.

## Case presentation

**Figure 1:**
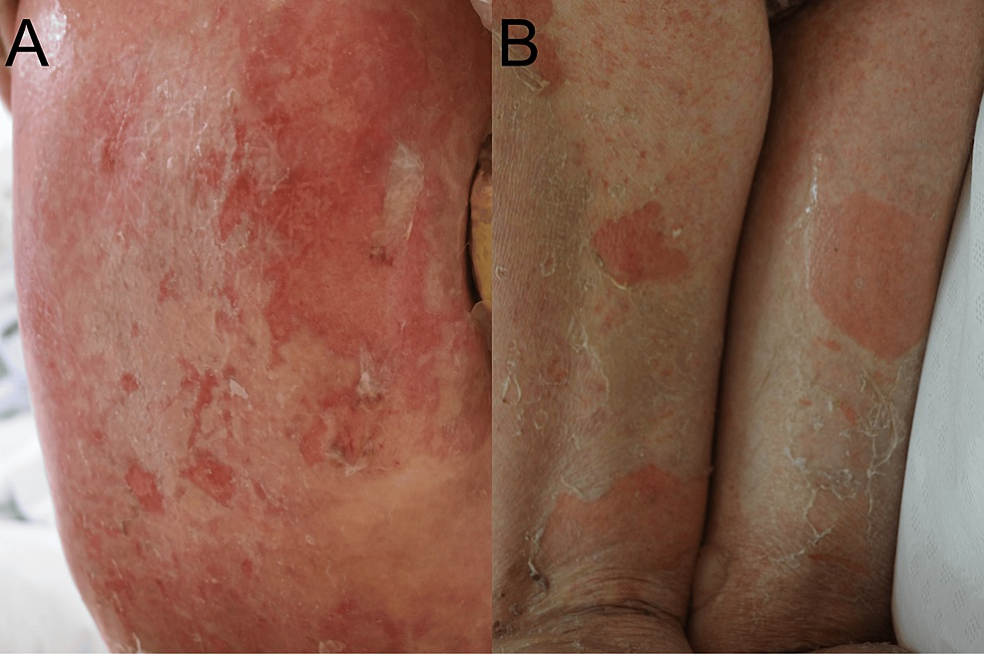
Clinical manifestations Erythematous plaques with erosions were observed on her back (A) and the back of the thigh (B).

A 63-year-old female with right breast cancer and distant lung metastasis received fluorouracil, epirubicin, and cyclophosphamide (FEC75) chemotherapy followed by nanoparticle albumin-bound paclitaxel (nab-PTX). However, her breast cancer was resistant to these treatments. Consequently, eribulin was administered. After the second dose of eribulin, she experienced a fever of 38°C and erythematous plaques, which gradually showed spontaneous improvement. Three days after the third eribulin dose, she developed erosions and widespread erythematous plaques over her body, including mucosal lesions. Physical examination showed extensive erythema with blisters and erosions on her trunk and limbs with mucosal lesions on her lips and genital area with fever over 38°C without hypotension (Figure 1A, 1B). This patient presents with extensive mucous membrane involvement, more than 10% of body surface area affected, and a positive Nikolsky's sign. Laboratory data revealed that anti-desmoglein 1/3 and anti-BP180 antibodies were negative. The white blood cell (WBC) count was 2,000/μL (normal range: 3,300-8,600/μL), with eosinophils making up 3% (normal range: 0%-8.5%) of the total. Blood urea nitrogen (BUN) levels were at 18 mg/dL (normal range: 8-20 mg/dL). The patient's body temperature (BT) was 37.6°C, and the heart rate (HR) was 131 beats per minute. Based on these findings, a Severity-of-Illness Score for Toxic Epidermal Necrolysis (SCORTEN) score of 4 was calculated, including factors such as age, body surface area involvement of more than 10%, the presence of comorbidity of malignancy, and a heart rate exceeding 120 beats per minute.

**Figure 2:**
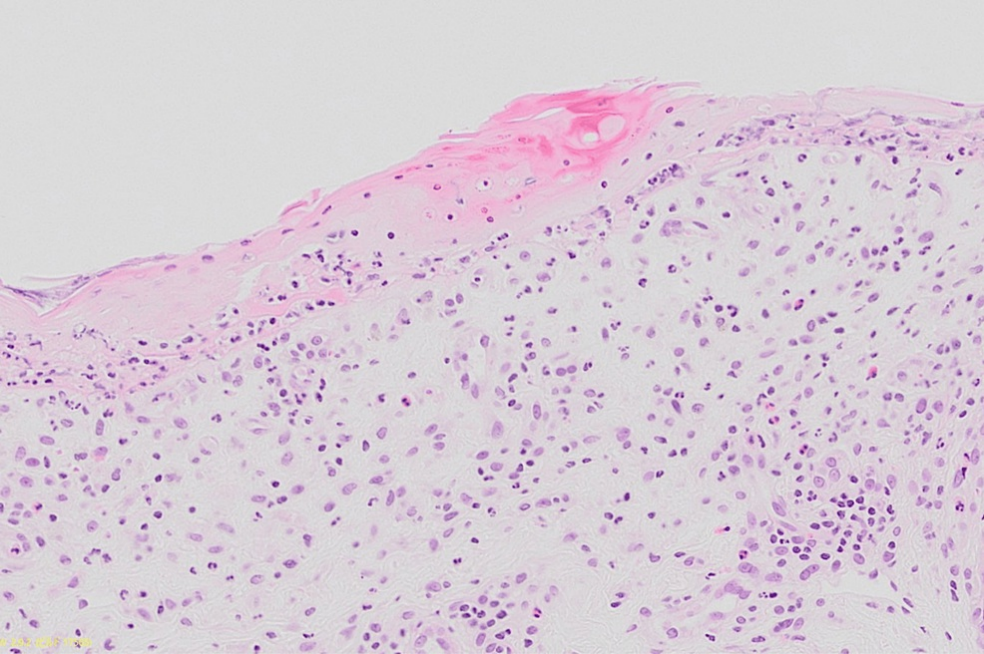
Histological examinations A skin biopsy taken from erythematous plaque revealed dyskeratotic keratinocytes in the epidermis with inflammatory cell infiltration in the dermis.

A skin biopsy taken from erythematous plaque revealed dyskeratotic keratinocytes in the epidermis with blister formation in addition to the inflammatory cell infiltration with eosinophils in the epidermis (Figure 2). Based on these findings, she was diagnosed with toxic epidermal necrolysis. Treatment with steroid pulse therapy with 1,000 mg of methylprednisolone per day for three days followed by oral prednisolone 50 mg (1 mg/kg) was started. One week later, she responded well to the steroids showing significant improvement in inflammation and nearly complete healing of erosions on her abdomen and distal limbs. Although the skin eruption reappeared after the third administration of eribulin, a lymphocyte transformation test for eribulin was conducted, yielding a stimulation index of 2.6 (Table 1). The test is performed by isolating lymphocytes from the patient's blood and exposing them to the drug in question to observe if they become activated, indicating a sensitivity. "CPM" stands for counts per minute, a measure of radioactive decay used to assess cell proliferation. The stimulation index is used to determine the level of activation compared to the control. Combined with in vitro results, these findings led to a diagnosis of toxic epidermal necrolysis caused by eribulin.

**Table 1:** Lymphocyte stimulation test CPM: counts per minute

Drug	CPM (unit)	Stimulation index
Control	540	1.0
Eribulin	1,416	2.6

## Discussion

Eribulin targets the polymerization of tubulin subunits, crucial components of the cellular microtubule network, which plays a vital role in cell division and structural integrity. Beyond its anti-tumor properties, eribulin has been observed to enhance the cytotoxic response of CD8-positive lymphocytes [3] and activate cyclic GMP-AMP synthase-stimulator of interferon genes (cGAS-STING)-mediated immune responses [4], thereby boosting overall cytotoxic immune activity [5,6]. These immunomodulatory effects, while beneficial in targeting cancer cells, may also predispose to atypical immune reactions such as the severe cutaneous adverse reaction seen in this case.

The rarity of reported cutaneous reactions in patients treated with eribulin might be attributed to its mechanism of action. Eribulin's anti-polymerization effect could potentially impair the delayed hypersensitivity reactions in antigen-presenting cells, which are essential for initiating cutaneous immune responses [7]. This impairment may typically reduce the likelihood of hypersensitivity reactions but does not preclude severe adverse effects as demonstrated by this case.

Given the critical role of CD8-positive lymphocytes in mediating severe drug eruptions and the observed immunological effects of eribulin, further research is essential. Detailed case studies and investigations are needed to better understand the mechanisms behind eribulin-mediated cutaneous adverse reactions and to develop strategies for preventing and managing such reactions in cancer patients undergoing treatment with this drug.

## Conclusions

This case marks the first reported instance of TEN associated with eribulin, a chemotherapy drug known for its microtubule-inhibiting properties. Despite the rarity of cutaneous adverse reactions with eribulin, this case emphasizes the importance of monitoring for severe drug eruptions. After discharge, the patient underwent monthly examinations for six months, during which there was no recurrence of the skin eruption. The occurrence of TEN in this context highlights the need for further research to understand eribulin's immunomodulatory effects and its potential to trigger significant dermatological reactions.
